# Efficient Adsorption of Sulfamethazine onto Modified Activated Carbon: A Plausible Adsorption Mechanism

**DOI:** 10.1038/s41598-017-12805-6

**Published:** 2017-09-29

**Authors:** Ying Liu, Xiaohui Liu, Wenping Dong, Lingli Zhang, Qiang Kong, Weiliang Wang

**Affiliations:** 1 College of Geography and Environment, Shandong Normal University, Jinan, 250358 PR China; 2 School of Environment, Tsinghua University, Beijing, 100084 PR China; 3 Shandong Academy of Environmental Science and Environmental Engineering Co., Ltd, Jinan, 250013 PR China; 4 Institute of Environment and Ecology, Shandong Normal University, Jinan, 250358 PR China

## Abstract

Activated carbon (AC) was modified by FeCl_3_. Batch experiments were carried out to evaluate the characteristics of equilibrium, kinetics and thermodynamics of Sulfamethazine adsorption onto original and modified AC. The results showed that Fe^3+^ treatment changed the surface area, pore volume and surface zeta potential and increased the number of surface oxygenic functional groups. The adsorption of Sulfamethazine on modified activated carbon (MAC) was significantly improved. Isotherm test results revealed that the adsorption isotherms of Sulfamethazine on MAC fit the Freundlich, Langmuir and Temkin equations well. The maximum adsorption quantity of Sulfamethazine on MAC was 17.2414 mg/g at 25 °C. The adsorption kinetics of Sulfamethazine on AC and MAC can be characterized by the pseudo-second-order model. The adsorption process was affected by membrane diffusion, surface adsorption and internal diffusion. The adsorption quantities of Sulfamethazine first increased and then decreased for pH between 3 and 10. The removal efficiencies decreased with increasing temperature, which is favorable for adsorption at low temperature. It was also found that the mechanisms of adsorption included micropore capture and electrostatic, hydrogen bonding, π-π electron donor-acceptor (EDA) and coordination interactions as well as other interactions.

## Introduction

Sulfonamides are emerging pollutants that are mainly used for the treatment of diseases caused by a variety of bacterial infections; they are widely used in animal husbandry and aquaculture^1^ and have attracted widespread research attention. Most sulfonamides are released into the environment through animal feces and urine in the form of the original drug or metabolites, which can exist for a long time in the environment, becoming a potential risk of environmental pollution^2^. Studies have shown that residues of these antibiotics can enter the soil, surface water, groundwater and even drinking water through farm runoff and urban sewage treatment plants^3–6^.

Sulfonamides slowly degrade after entering the environment. The residual concentration of the antibiotic pollutants is only within ng/L-μg/L in environmental water, but it accumulates in the human body and will ultimately endanger human health when humans are exposed to low concentrations for a long time^7,8^. Additionally, excess antibiotics and metabolites can induce microbial resistance genes, which can also cause potential risk to the ecological environment and human health even if their exposure in the environment is limited to trace levels. Additionally, Sulfamethazine can cause acute or chronic poisoning at a lower exposure level.

Traditional wastewater treatment technology can only remove some antibiotics from wastewater^9–11^. This has motivated researchers to develop new technologies that are simple and more efficient for effective removal of sulfonamides in wastewater. Activated carbon can effectively remove chroma, odor, and some inorganic compounds and most organic contaminants^12^, due to its large specific surface area and complex pore structure. However, its use in practical applications has been limited by its lower adsorption efficiency and cost constraints. Metal ion modification^13^ is a common method for the modification of activated carbon that is simple, inexpensive, and can significantly improve the adsorption performance of activated carbon. However, the adsorption mechanisms of sulfonamides on activated carbon are not clear at present, and the related relationship between the adsorption capacity of sulfonamides and their physical and chemical properties is currently poorly understood.

In this study, Fe^3+^ was adopted to modify activated carbon. Batch adsorption experiments were used to explore the adsorption mechanism. The adsorption characteristics of Sulfamethazine on original and modified activated carbon were investigated by adsorption kinetics, adsorption thermodynamics, and adsorption isotherms, which can provide the scientific basis for the removal of pharmaceuticals and personal care products (PPCPs) from wastewater.

## Materials and Methods

### Materials and chemicals

Sulfamethazine (SMX ≥99.0%) was used as an adsorbate compound and was received from Dr. Ehrenstorfer, Germany. The chemical structures and physico-chemical properties of Sulfamethazine are presented in Table 1. Methanol and formic acid were used as the chromatographic reagent, and other chemicals were used as analytical reagents. Water used in the study was purchased from Hangzhou Wahaha Group Co. Ltd.

**Table 1:** Physic-chemical properties of Sulfamethazine.

Compound	CAS	Chemical formula	M(g·mol^−1^)	Melting point(°C)	pK_a_	Solubility(mg/L)	logK_ow_
SMX	57-68-1	C_12_H_14_N_4_O_2_S	278.33	176	2.28	1500	0.89
					7.42		

Activated carbon (AC) was purchased from an activated carbon factory in Jiangsu. It was screened through a 100-mesh sieve to obtain a homogenous powder, scrubbed 5 to 6 times, and then dried for 8 h at 105 °C.

### Preparation of modified activated carbon

A total of 10 g AC was mixed with 100 mL of a 0.75 mol·L^−1^ FeCl_3_ solution using a 250-mL conical flask equipped with polytetrafluoroethylene-lined screw caps. It was shaken in a thermostatic oscillator at a constant agitation speed (180 rpm) for 24 h. After centrifugation was used to separate the liquid from the solid, the solid was extracted and filtered with a 0.45-μm cellulose ester membrane filter and cleaned with deionized water 5–6 times. Then, it was dried at 105 °C for 8 h and ground. Finally, modified activated carbon (MAC) was obtained by passing through a 100-mesh sieve.

### Batch Adsorption Experiments

Batch adsorption experiments for Sulfamethazine were conducted at different adsorption times, initial adsorbate concentrations, pH, and temperatures according to OECD guideline 106^14^. The calibration curves (0.1–100 mg·L^−1^ concentrations) for analyte detection presented good linearity (R^2^ > 0.999). Samples of the SMX solutions without the solid particles were kept under the same conditions as the blank sample. The natural SMX degradation rate in the blank sample was less than 1% and was ignored. In addition, the coefficient of variance for the UPLC analysis was less than 5% based on three measurements.

### Adsorption isotherms

A total of 0.15 g AC and MAC was mixed with 25 mL varying initial SMX concentrations (10–100 mg·L^−1^) using 30-mL glass vials equipped with polytetrafluoroethylene-lined crew caps. The SMX solutions with 0.01 mol·L^−1^ CaCl_2_ were shaken in a thermostatic oscillator at a constant agitation speed (220 rpm) for 24 h at an unadjusted pH. Centrifugation of the SMX solutions at 5000 rpm for 15 minutes was used to separate the liquid from the solid. Finally, the supernatant solution was extracted and filtered with a 0.22-μm PTFE microporous membrane.

### Adsorption kinetics

The adsorption kinetics study of AC and MAC was performed by adding 0.15 g sample and 20 mL 100 mg·L^−1^ SMX solutions to 30-mL glass vials. Then, all glass vials were shaken at a constant agitation speed (220 rpm) at 25 °C. Samples were obtained at 5, 10, 20, 40, 60, 90, 120, 180, 360, 540, 720, 1080, 1440, 2160, and 2880 min, respectively.

### Effect of pH on the adsorption equilibrium

A total of 0.15 g AC and MAC added to 25 mL SMX solution in a 30-mL glass vial at 25 °C was shaken in a thermostatic oscillator at a constant agitation speed (220 rpm) for 24 h. The aqueous solution of 0.01 mol·L^−1^ CaCl_2_ containing the test Sulfamethazine at the 100 mg·L^−1^ concentration was preadjusted for pH values between 3 and 10 with 0.01 mol·L^−1^ NaOH and HCl.

### Detection of antibiotics

The concentrations of Sulfamethazine in the supernatants were analyzed by HPLC (Agilent Technologies 1200 Series) using a 4.6 × 250 mm Athena C18-WP column and UV detector. The mobile phase for Sulfamethazine quantification was 0.1% formic acid/methanol (65%:35%, v:v), and the wavelength of the UV detector was 265 nm. Methanol and 0.1% formic acid were filtered by the microporous organic membrane (0.22 μm) and the microporous drainage membrane (0.22 μm) before use. The flow rate was 0.8 mL/min, and the column temperature was 30 °C. The injection volume was 20 μL.

### Characterization

A specific surface area analyzer (BET, SSA-4000, Beijing) was used to determine the specific surface area, and pore volume and aperture of the original and modified activated carbon samples. The surface morphology, pore structure and elements analyses of AC and MAC were performed using SEM-EDS (JSM-6700F, Japan) and TEM (JEM-2100, Japan), respectively. The graphitizing level of adsorbent was analyzed using Raman spectroscopy (Renishaw-RM2000). XRD (Bruker D8) was used to the crystal form of AC and MAC.

### Data availability statement

The authors agree that the data is available.

## Results and Discussion

### Characteristics of adsorbents

#### Chemical and physical properties

The chemical structures and physico-chemical properties of the original and modified activated carbon are presented in Table 2. The surface area, total pore volume, micropore volume and mesopore volume of activated carbon all increased to some extent after modification by Fe^3+^, which can provide more adsorptive sites for Sulfamethazine. The microporous structure of the activated carbon could be broken because of the modification, giving rise to the larger specific surface area. Fe^3+^ is mainly deposited in the mesopores during the activation process, enlarging the pore size and entering the micropores. The oxidation gas released by the metal salt was introduced into the micropore and reacted with the microporous carbon wall^15^.

**Table 2:** The chemical and physical properties of original and modified activated carbon.

Materials	BET m^2^/g	Average pore size nm	Total porous volume cm^3^/g	Micropore volume cm^3^/g	Mesopore volume cm^3^/g
AC	70.96	2.90	0.1029	0.0310	0.0719
MAC	166.23	4.76	0.192539	0.072302	0.1202

**Figure 1:**
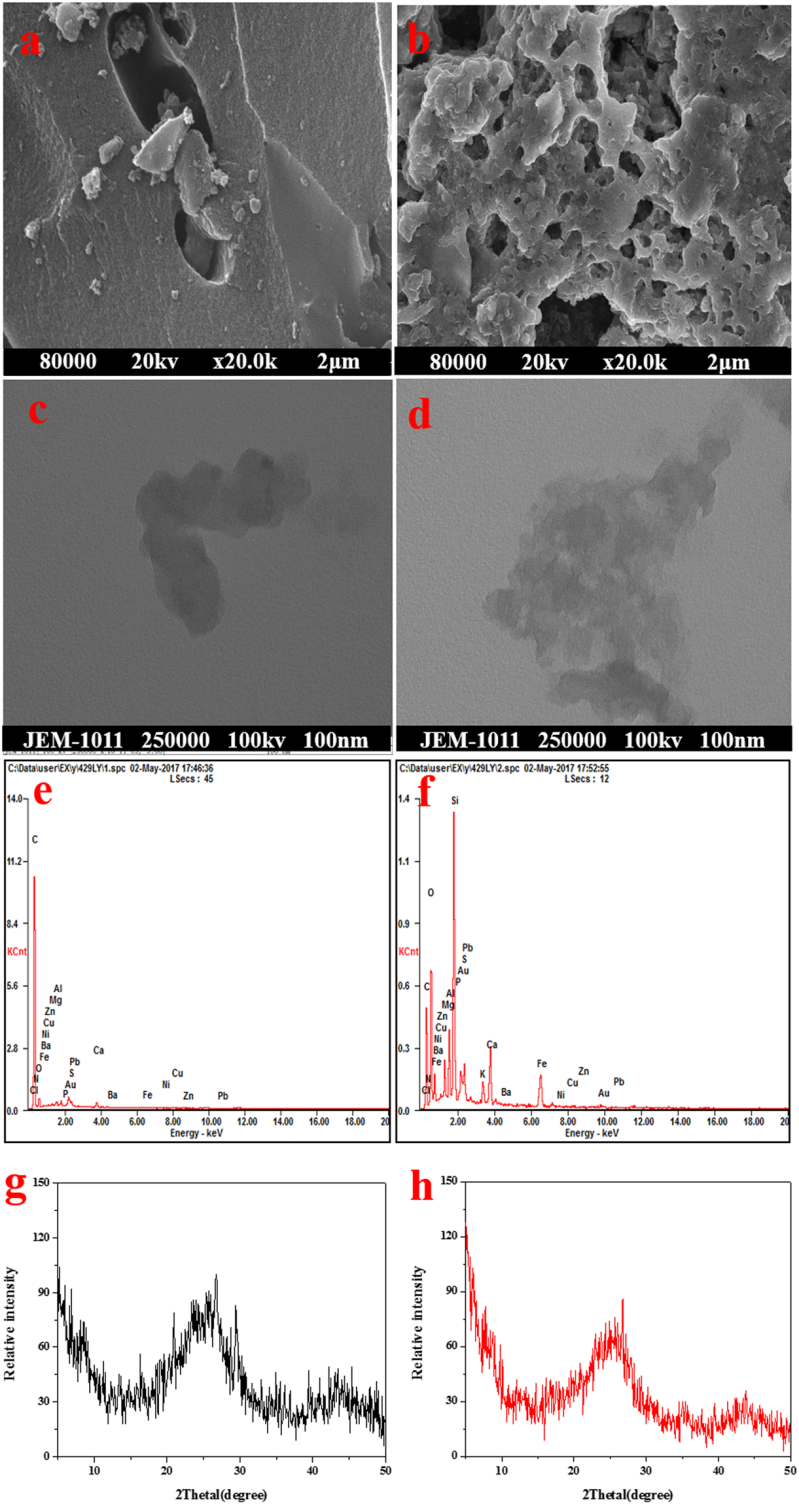
Representative SEM, TEM and EDS images of two adsorbents. (**a**) SEM of AC (scale bar is 2 μm), (**b**) TEM of AC (scale bar is 100 nm), (**c**) EDS of AC, (**d**) SEM of MAC (scale bar is 2 μm), (**e**) TEM of MAC (scale bar is 100 nm), (**f**) EDS of MAC, (**g**) XRD of AC, (**h**) XRD of MAC.

The SEM images of AC and MAC are shown in Fig. 1(a) and (b). The AC morphology suggests a smooth surface with regular pore structure. Compared to natural activated carbon, the surface of MAC appears coarse and irregular. The regular pore structure was destroyed, and the porosity was increased. This may be due to Fe^3+^ introduced in the internal pores of the activated carbon, resulting in the oxidation of the carbon wall and an increase in the pore size. The coarse surface structure and porous characteristics are conducive for the acceleration of the spread of Sulfamethazine in the activated carbon. TEM images of AC and MAC are shown in Fig. 1(c) and (d). The photographs suggest that AC is amorphous. Compared to AC, MAC exhibits many pores and transparent spots, further suggesting that the surface area increased. The EDS images of AC and MAC are shown in Fig. 1(e) and (f). Compared to AC, the elemental O and Fe content of MAC was significantly increased, and the C content was decreased, indicating that Fe^3+^ was loaded on the activated carbon. Additionally, it is further proved that surface oxygenic functional groups were increased, which is favorable for the adsorption of Sulfamethazine. The XRD patterns of AC and MAC are shown in Fig. 1(g) and (h). And there is not the distinct characteristic peaks of AC and MAC, showing that they are amorphous. This is consistent with the results of TEM photographs.

#### FT-IF spectra

The adsorption capacity of sulfonamides on modified activated carbon was greatly altered due to the change of the surface chemical properties and the modification of the pore structure. The changes in the surface chemical properties can be determined by the analysis of functional groups on the original and modified activated carbon.

**Figure 2:**
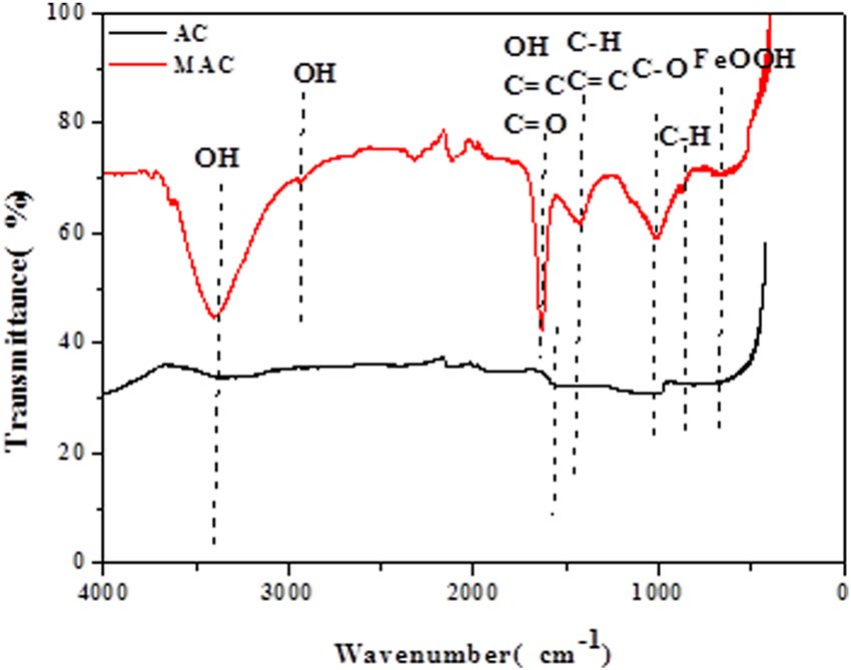
FT-IR spectra of original and modified activated carbon.

FT-IR spectra of original activated carbon and that modified by Fe^3+^ are shown in Fig. 2. There are the absorption peaks on the original and modified activated carbon at 3400 cm^−1^ that are due to the -OH vibrations associated with hydrogen bonding, phenolic hydroxyl and N-H stretching vibrations. The intensity of the –OH peak is significantly enhanced, indicating that the number of -OH is increased by the modification. Compared to AC, there is a significant adsorption peak for MAC at 2850 cm^−1^. This may correspond to the OH stretching vibration. The band at 1630 cm^−1^ could be the N-H deformation vibration, phenolic hydroxyl, C=C or C=O stretching vibration. It is obvious that the absorption spectra exhibit a redshift. The bands at 2120 cm^−1^ and 1010 cm^−1^ can be assigned to the N-H and C-O stretching vibrations, respectively. The band at 665 cm^−1^ can be assigned to the FeOOH stretching vibration, which is the typical group of the activated carbon modified by Fe^3+^. These groups can further improve the adsorption ability of activated carbon.

The FT-IR spectra of original and modified activated carbon exhibit similar adsorption peaks, indicating that original and modified activated carbon possess similar chemical properties. However, the absorbance of MAC is significantly decreased, demonstrating that the organic structures of activated carbon were destroyed, and the acidic group content was increased during the modification process. Additionally, the number of active sites for sorption of Sulfamethazine was increased on the modified activated carbon.

#### Raman spectra

**Figure 3:**
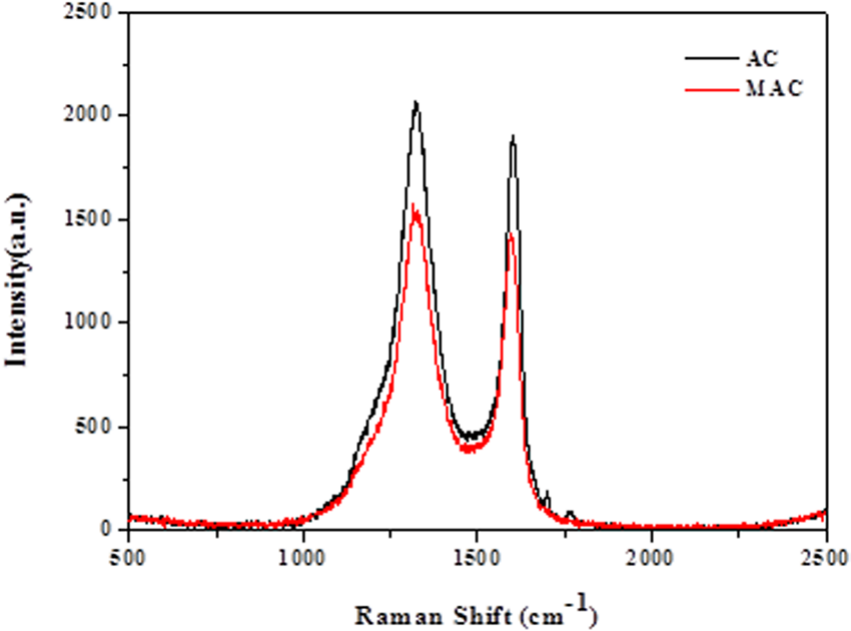
Raman spectra of original and modified activated carbon.

Two peaks are observed in the Raman spectra of carbon materials (Fig. 3), with the first peak near 1595 cm^−1^ (G peak), which is the characteristic scattering peak of graphite, and the second peak at 1350 cm^−1^ (D peak), which is caused by lattice defects, disordered arrangement and the low symmetry carbon structure of graphite^16^. The degree of graphitization can be evaluated using the ratio of intensities of the n D peak and G peaks:

1 $${\rm{R}}=\frac{{I}_{D}}{{I}_{G}}$$

where R is the degree of graphitization of carbon materials, ${I}_{D}$ is the intensity of the D peak at 1360 cm^−1^, and ${I}_{G}$ is the intensity of the G peak at 1580 cm^−1^. A smaller R value corresponds to a higher degree of graphitization^17^. The values of the original and modified activated carbon were 1.11 and 1.06, respectively, indicating that the degree of graphitization of the Fe^3+^-modified activated carbon was higher, and to some extent, it can promote the adsorption ability of Sulfamethazine.

#### Effect of pH

The pH value has a great impact on the adsorption of Sulfamethazine. The pH of a solution controls the dissociation degree of the surface functional groups on the adsorbent, which can change the surface potential of activated carbon^18^. The chemical functional groups on activated carbon depend on the solution pH. When the pH value reaches a certain range, the functional groups, such as hydroxyl and carboxyl, will be hydrolyzed and affect the surface charge on the activated carbon. The pH value of the solution can also influence the solubility and form of Sulfamethazine. Thus, pH will affect the adsorption process.

The forms in which Sulfamethazine exists were different at different pH values, and its dissociation constants were 2.28 (pK_a,1_) and 7.42 (pK_a,2_). Depending on pH, Sulfamethazine can assume either a cationic, neutral or anionic form^18^. High adsorption capacities were achieved at pH values between pK_a,1_ and pK_a,2_, where Sulfamethazine almost completely exists in the neutral form, indicating that neutral Sulfamethazine is easily adsorbed onto activated carbon compared to that in the cationic or anionic form. With decreasing pH, the proportion of neutral Sulfamethazine decreased and that of anionic Sulfamethazine gradually increased. A large number of hydrophobic and hydrophilic groups are present on the surface of activated carbon, and Sulfamethazine can be adsorbed by hydrophobic effect with these groups. Sulfamethazine is deprotonated at a pH above 7.42, and is then present as a negative ion in solution. Due to the electrostatic repulsion between Sulfamethazine and the negative charge of activated carbon, the adsorption of Sulfamethazine is decreased.

**Figure 4:**
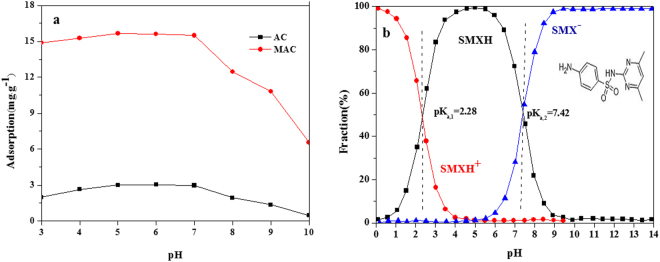
Effect of pH on the removal of Sulfamethazine (**a**) the adsorption of SMX, (**b**) the mole fraction of cationic (diamonds), neutral (squares), anionic (triangles).

The adsorption of Sulfamethazine on AC and MAC first increased and then decreased for pH values between 3 and 10 (Fig. 4). When the pH was below 5.0, adsorption was increased. This may be due to the coexistence of a competitive adsorption with H^+^, resulting in lesser adsorption. With increasing pH, competitive adsorption decreased and Sulfamethazine adsorption increased. Due to the weak deprotonation of amidogen on Sulfamethazine, for a pH between 5.0 and 7.0, Sulfamethazine exists almost completely as a protonated neutral molecule. The electron of benzene can be attracted by the amidogen and sulfonyl of neutral Sulfamethazine, which makes Sulfamethazine a π electron acceptor, thus enabling greater adsorption.

The adsorption of Sulfamethazine on MAC is higher than that on AC. This may be due to the coordination between the Fe ion and Sulfamethazine. The proportion of anion Sulfamethazine will be increased at pH above 8.0, increasing the electrostatic repulsion and inhibiting the hydrophobic interaction between neutral Sulfamethazine and activated carbon. Additionally, Fe is mainly in the ionic state on the surface of modified activated carbon under alkaline conditions, resulting in weak coordination ability. At the same time, the carboxylic acid on the surface of the modified activated carbon is changed into the amidogen, where the electrostatic repulsion with amidogen was increased and the adsorption capacity decreased.

#### Adsorption isotherms

**Figure 5:**
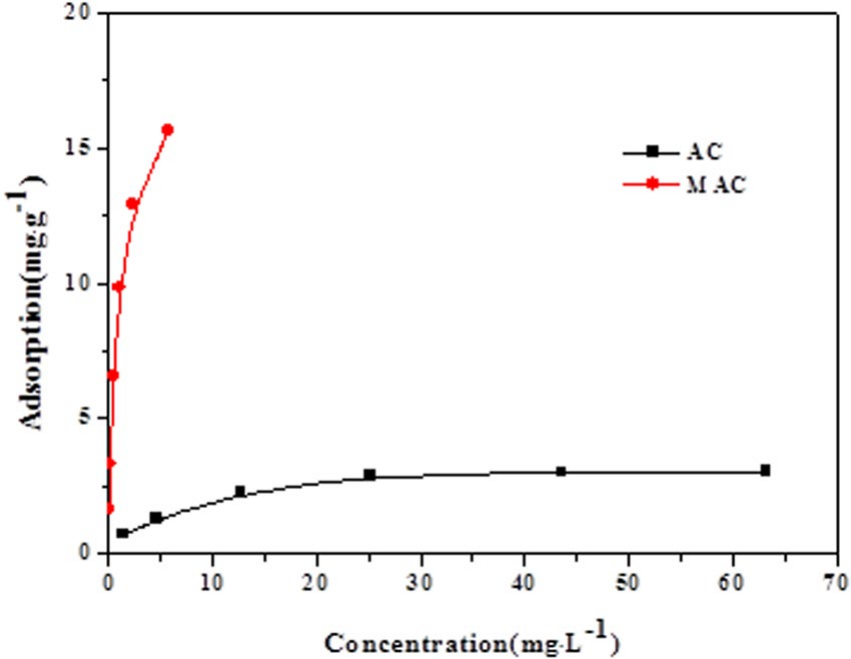
Adsorption isotherms of SMX on AC and MAC at different temperature.

The adsorption isotherms of AC and MAC at 25 °C are presented in Fig. 5. The adsorption of Sulfamethazine rapidly increased when the equilibrium concentration of SMX was low. As the SMX equilibrium concentration increased, the adsorption rate gradually decreased, and then reached a saturation state.

The amount of the adsorption of the unmodified activated carbon is lower from Fig. 5, and the maximum adsorption amount was 3.0713 mg/g at 25 °C. However, the adsorption amount on activated carbon modified with Fe^3+^ was significantly improved, and the maximum adsorption value was 17.2414 mg/g at 25 °C, showing an increase by a factor of 5.6. Sulfonamides are polar organic compounds, and a large number of functional groups was introduced to the surface of activated carbon by Fe^3+^ modification, increasing the surface polarity of activated carbon. Many hydroxyl and carboxyl groups are present on the surface of the modified activated carbon that can react with alkaline amidogen of Sulfamethazine to form ionic bonds. The Fe^3+^ ion on the surface of the modified activated carbon can coordinate with amidogen, which can improve the adsorption ability of Sulfamethazine.

Three different isotherm models were employed to fit the adsorption equilibrium isotherm. The equations of Langmuir, Freundlich, and Temkin were expressed as the following:

Langmuir model:

2 $${q}_{e}=\frac{{K}_{L}{q}_{max}{C}_{e}}{1+{K}_{L}{C}_{e}}$$

Freundlich model:

3 $${q}_{e}={K}_{F}{{C}_{e}}^{1/n}$$

Temkin model:

4 $${q}_{e}={K}_{T}ln{C}_{e}+{K}_{T}lnf$$

where q_e_ (mg/g) is the adsorption amount of SMX adsorbed per unit mass of the adsorbent; C_e_ (mg/L) is the adsorption equilibrium concentration of SMX; q_max_ (mg/g) is the maximum adsorption capacity that depends on the properties of the adsorbent; K_L_ (L/mg) is the Langmuir adsorption affinity constant related to adsorption bond energy; K_F_ and 1/n are the Freundlich adsorption constants representing the adsorption capacity and the adsorption intensity, respectively; 1/n represents the nonlinear degree of adsorption and the difference in the adsorption mechanism, and it is generally considered that preferential adsorption occurs when n > 1; and K_T_ (L/kg) is the Temkin adsorption constant for balanced binding energy.

The Langmuir isotherm can be expressed by means of a separation factor (${R}_{L}$), and is calculated using the following equation:

5 $$\,{R}_{L}=\frac{1}{1+{K}_{L}{C}_{0}}$$

where C_0_ (mg/L) is the initial concentration of SMX. Several types of adsorption isotherms can be characterized by R_L_. While R_L_ = 0, the adsorption process on the adsorbent is irreversible. When the R_L_ values lie between 0 and 1, the adsorption process is favorable, and this is the optimal adsorption isotherm. Linear adsorption is expressed as R_L_ = 1. Otherwise, it is difficult for adsorption on an adsorbent to occur.

Table 3 shows the data fitted with the isothermal adsorption models for Sulfamethazine. The R^2^ values obtained using the Langmuir and Temkin models are larger than that of the Freundlich model, suggesting that these models can better fit the adsorption process of Sulfamethazine on the original and modified activated carbon. However, compared to the Freundlich model, the Langmuir and Temkin model can fit the adsorption process well, which showed that adsorption of Sulfamethazine was dominated by monolayer adsorption and surface adsorption. In the present study, the R_L_ values were all between 0 and 1, suggesting that the adsorption was favorable. As the interaction between Sulfamethazine and modified activated carbon increases, the adsorption energy linearly decreased^19^.

**Table 3:** Parameters for adsorption isotherm of Sulfamethazine on AC and MAC at different temperature.

		Langmuir				Freundlich			Temkin	
		q_max_	K_L_	R_L_	R^2^	K_F_	1/n	R^2^	K_T_	R^2^
		(mg·g^−1^)	(L·mg^−1^)			(mg·g^−1^) (L·mg^−1^)^1/n^				
AC	298 K	3.0713	0.2169	0.0461	0.9764	0.7005	0.3968	0.9499	0.6738	0.9668
MAC	298 K	17.2414	1.6910	0.0059	0.9776	8.2604	0.4824	0.9420	3.1358	0.9960

The Freundlich constant 1/n was below 0.5, suggesting that the preliminary adsorption process easily occurs. Additionally, n > 1, indicating that the adsorption of Sulfamethazine is preferential on the original and modified activated carbon. With increasing Sulfamethazine concentration, the adsorption process becomes difficult. The Langmuir coefficient R_L_ values were all between 0 and 1, suggesting that the adsorption of Sulfamethazine is also preferential^20^, and further proving that the Langmuir model can fit the adsorption process well.

Previous studies of sulfonamides have mainly focused on soil, humus, organic materials and clay minerals. Comparative analysis for adsorption on the materials investigated in recent years is shown in Table 4. The adsorption ability of Sulfamethazine on Fe^3+^-modified activated carbon was higher than that on other adsorbents. Fe^3+^-modified activated carbon has the advantages of being simple, economical, and environmentally friendly, and its low cost, mild preparation conditions, and high removal efficiency of Sulfonamide antibiotics will lead to growing attention for its use in practical applications.

**Table 4:** Comparison of Sulfamethazine adsorption capacity of different adsorbents.

adsorption material	qmax	References
activated carbon	3.0713 mg/g	this research
activated carbon modified by Fe^3+^	17.2414 mg/g	this research
high silica zeolite	1.00 × 10^−3^ mol/g	18
magnetic nanocomposite CoFeM48	68.9 μg/g	20
paddy soil	49.3 mg/kg	21
organophilic zeolite Y	777 μmol/g	22

#### Adsorption kinetics

**Figure 6:**
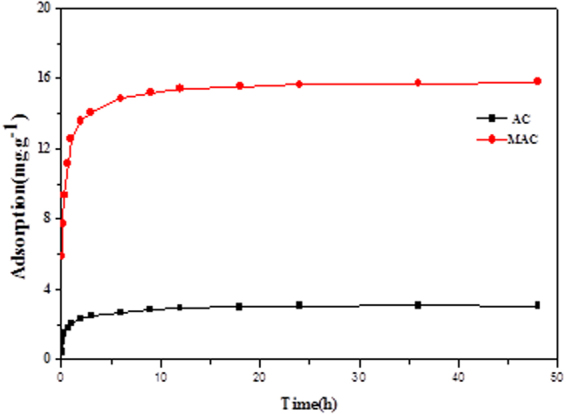
Adsorption kinetics of Sulfamethazine on AC and MAC.

Adsorption kinetics are mainly used to study the changes in the adsorption rate with time. The adsorption kinetics curves of the original and modified activated carbon at 25 °C show similar adsorption features (Fig. 6) and can both reach equilibrium in 24 h. The adsorption amounts increased with time; the adsorption process of two materials is at the fast reaction stage (0–2 h) at first, and the adsorption quantities were 76.2% and 86.9% of the equilibrium adsorption quantity (3.04 mg/g and 15.64 mg/g), respectively. The adsorption capacity is linear with time, and then slows when the surface active sites of the original and modified activated carbon are occupied. The adsorption process of Sulfamethazine can be divided into two processes – the fast adsorption stage and the slow adsorption stage. The dispersion of activated carbon or Sulfamethazine was high in water in the fast reaction stage, and Sulfamethazine was rapidly diffused into the active sites of activated carbon. Then, Sulfamethazine was diffused into internal pores of activated carbon, increasing the mass transfer resistance with time in the slow reaction stage. The adsorption rate depended on the adsorbent properties and solution pH and slowed down with time to reach equilibrium.

To explore the characteristics of adsorption kinetics, the current study used three kinetic models to fit the process of Sulfamethazine adsorption on original and modified activated carbon. The pseudo-first-order, pseudo-second-order and intraparticle diffusion equations^21,22^ are expressed as follows:

Pseudo-first-order model:

6 $${ln}({q}_{e}-{q}_{t})=ln{q}_{e}-{k}_{1}t$$

Pseudo-second-order model:

7 $$\frac{t}{{q}_{t}}=\frac{1}{{k}_{2}{{q}_{e}}^{2}}+\frac{t}{{q}_{e}}$$

Intraparticle diffusion model:

8 $${q}_{t}={k}_{3}{t}^{0.5}+C$$

where q_e_ and q_t_ (mg/g) are the amounts of SMX adsorbed per unit mass of the adsorbent at equilibrium and at time t (h), respectively; k_1_ and k_2_ (g/mg·min^−1^) are the pseudo-first-order and pseudo-second-order adsorption rate constants, respectively; k_3_ (mg/(g·min^1/2^)) is the adsorption rate constant of the intra-particle diffusion model related to the particle diffusion coefficient D, ${k}_{3}=\frac{6{q}_{e}}{r}\sqrt{\frac{D}{\pi }}$, and r is the particle radius.

Table 5 shows the data fitted with the mass transfer model. The pseudo-second-order model exhibited better fitting of the adsorption process on the original and modified activated carbon, and the correlation coefficient of R^2^ > 0.999 suggested that the adsorption rate was proportional to the square of the pollutant concentration. The chemical reaction may be an important limiting factor of the adsorption process, and it can also be affected by the interaction between adsorbent and adsorbate and exchange electrons^23^. The pseudo-first-order kinetic equation is more suitable to describe the dynamics of the initial stage, which have some limitations in the application process, while the pseudo-second-order model kinetic equation contains the complete three stages of the adsorption process—membrane diffusion, surface adsorption and internal diffusion—and can be more suitable for this adsorption process^24^.

**Table 5:** Fitting parameters of adsorption kinetics model of Sulfamethazine on AC and MAC.

	Pseudo first-order model			Pseudo second-order model			Intraparticle diffusion model	
	k_1_	q_e_	R^2^	k_2_	q_e_	R^2^	K_3_	R^2^
	min^−1^	mg/g		g/mg·min^−1^	mg/g		mg/g·min^0.5^	
AC	0.4732	3.168	0.9672	1.7244	3.098	0.9998	0.3128	0.6659
MAC	0.1363	14.82	0.9688	3.3743	15.85	1	1.1747	0.6062

**Figure 7:**
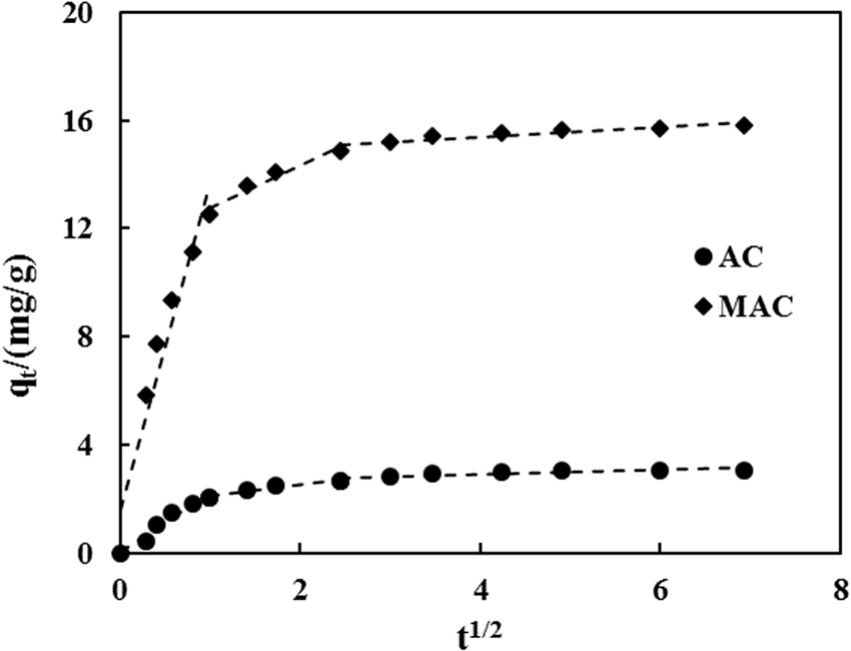
Intra-particle diffusion result of sorption of Sulfamethazine onto Activated Carbon.

To further identify the diffusion mechanism of the adsorption process of Sulfamethazine, the intraparticle diffusion model was used to determine the main controlling factors of the adsorption rate in the adsorption process. The results are expressed as follows (Fig. 7).

The adsorption and diffusion processes of Sulfamethazine on the original and modified activated carbon can be described with three stages as shown in Fig. 7. The adsorption rate is initially high due to adsorption on the surface of activated carbon at the first stage. Then, a rapid intraparticle diffusion process occurs where Sulfamethazine is gradually adsorbed onto activated carbon, and the adsorption rate constant is gradually decreased. At the third stage, the effects of the boundary layer and the mass transfer resistance increase, resulting in the slowing of intraparticle diffusion. The fitting equations for all three stages do not pass through the origin of the coordinates, suggesting that intraparticle diffusion is not the only step that controls the adsorption rate. Therefore, the adsorption process is also affected by the effects of membrane diffusion and surface adsorption.

#### Adsorption thermodynamics

**Figure 8:**
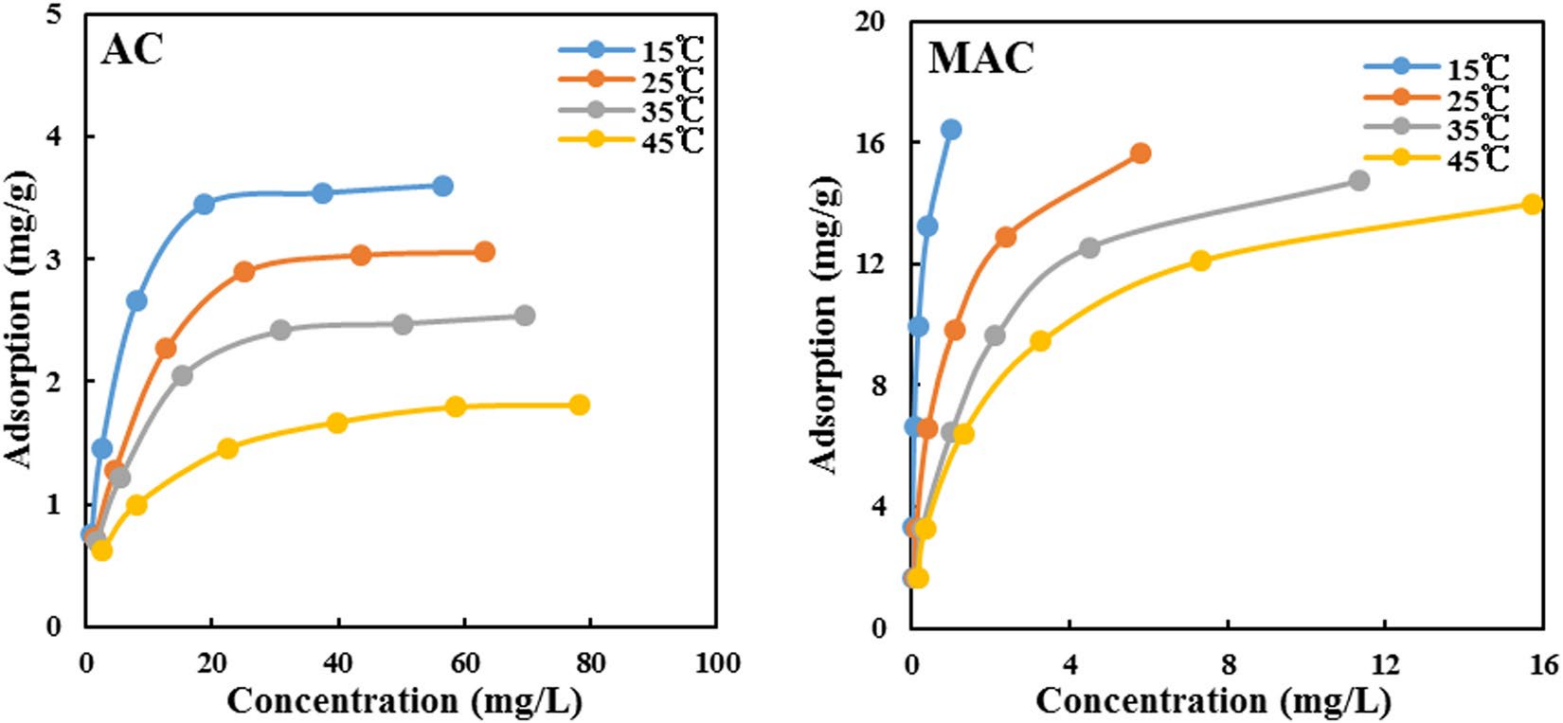
Adsorption amount of SMX under different temperature conditions.

The results for the adsorption of Sulfamethazine on the original and modified activated carbon at different temperatures are presented in Fig. 8. With increasing temperature, the adsorption amounts of Sulfamethazine on two activated carbon surfaces gradually decreased, indicating that high temperature was unfavorable for the adsorption reaction. Therefore, this showed that the adsorption process of the original and modified activated carbon was exothermic, and the adsorption of Sulfamethazine is more favorable at lower temperature.

The effects of temperature on adsorption can be expressed by thermodynamic parameters. The change of the adsorption free energy is an important parameter that reflects the adsorption characteristics of the adsorbent. When the free energy change is less than 40 KJ·mol^−1^, physical adsorption occurs; otherwise, chemical adsorption is present (State Environmental Protection Administration Toxic Chemicals Management Office, 1992). The values can be calculated using thermodynamic equations and adsorption constants, and the results are shown in Table 6.

**Table 6:** Thermodynamic parameters of Sulfamethazine adsorption on AC and MAC.

Adsorbent	T K	R_L_	∆G kJ·mol^−1^	∆H kJ·mol^−1^	∆S J·K^−1^·mol^−1^
AC	288	0.0350	−20.01	−17.03	10.38
	298	0.0461	−20.14		
	308	0.0476	−20.24		
	318	0.0511	−20.32		
MAC	288	0.0010	−23.49	−8.88	50.99
	298	0.0059	−24.17		
	308	0.0107	−24.65		
	318	0.0115	−25.02		

The effect of temperature on adsorption can be expressed by thermal characteristic parameters:

9 $${\rm{\Delta }}G=-RTln{K}_{t}$$

10 $$\mathrm{ln}\,{K}_{t}=\frac{{\rm{\Delta }}S}{R}+\frac{-{\rm{\Delta }}H}{RT}$$

where ΔG (kJ/mol) is the standard Gibbs free energy, and ΔG < 0, indicating that the reaction is spontaneous; Δ*S* (J/(K·mol)) and Δ*H* (kJ/mol) are changes of entropy and enthalpy, respectively; K_t_ and R (8.314 J/(mol·K)) are the Langmuir constant at temperature T (K) and gas constants, respectively. Δ*S* > 0 shows that the degree of disorder in the solid-liquid interface is increased. Δ*H* > 0 indicates that the adsorption process of the adsorbent is endothermic; otherwise, it is exothermic.

The standard Gibbs free energy (ΔG) was negative at the experimental temperature (Table 6), demonstrating that the adsorption process of Sulfamethazine on the original and modified activated carbon is spontaneous at temperatures between 288 K and 318 K, and |ΔG| < 40 kJ·mol^−1^, suggesting that Sulfamethazine adsorption on activated carbon is mainly of the physical adsorption type due to hydrogen bonding, hydrophobic, π-π conjugate, and coordination interactions^25^. ∆H < 0 shows that the adsorption process is exothermic, and higher temperatures are not favorable for the adsorption of Sulfamethazine. The relationship between R_L_ and temperature is presented in Table 6. All R_L_ values are below 1, suggesting that the adsorption process was spontaneous and favorable. With temperature increases, R_L_ increased, indicating that a decrease in the temperature could promote the adsorption reaction. ∆S > 0 suggests that the degree of disorder is increased in the adsorption process. There are many water molecules desorbed from activated carbon, resulting in a higher number of exposed adsorption sites on the surface of activated carbon.

#### Discussion of adsorption mechanism

**Figure 9:**
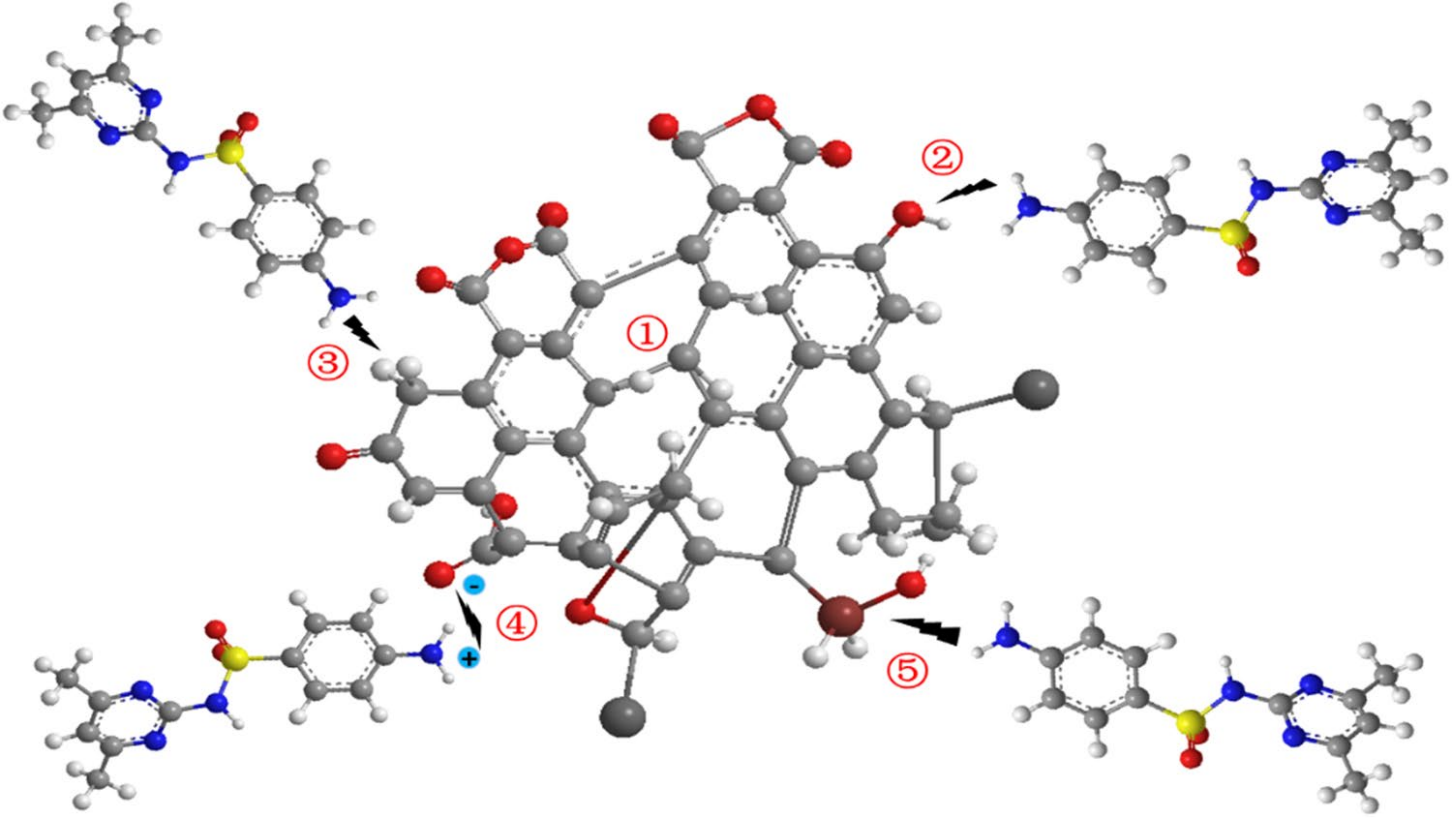
Schematic illustrations of plausible interaction between the Sulfamethazine and MAC. 1) Micropore capture 2) Hydrogen bonding interaction 3) π-π EDA interaction 4) Electrostatic interaction 5) Coordination interaction.

The surface of modified activated carbon shows many pores and transparent spots, and a large number of oxygen-containing functional groups are modified, resulting in a change in the Sulfamethazine adsorption mechanism. According to the related research results in this study, the adsorption mechanism of Sulfamethazine on the surface of activated carbon is summarized as follows (Fig. 9):

1. Micropore capture
  SEM images show that the regular pore structure of activated carbon was destroyed and porosity was increased. Additionally, TEM images show that the surface of modified activated carbon contains many transparent spots, indicating that the pore structures are more developed and more adsorption sites are available after the modification. This is conducive for the adsorption of Sulfamethazine into the pores of the black carbon molecular layer. Additionally, the surface area, total pore volume, micropore volume and mesopore volume of activated carbon were all increased to some extent after modification by Fe^3+^.
2. Hydrogen bonding interaction
  Oxygen-containing functional groups identified by FTIR spectra promoted Sulfamethazine adsorption by facilitating the hydrogen bond interactions between MAC surface and Sulfamethazine. It is clear from Fig. 2b that broad absorption peaks at 3400 and 2850 cm^−1^ indicate the presence of -OH on the MAC surface. These intermolecular hydrogen bonds enhanced the surface interactions between Sulfamethazine and MAC, which is expected to contribute to the excellent Sulfamethazine adsorption affinity of the MAC^26^.
3. π-π electron donor-acceptor (EDA) interaction.

The pH of Sulfamethazine is approximately 5.0–7.0, where the amidogen on Sulfamethazine is protonated. The electron of the benzene ring can be attracted by the amidogen and sulfonyl of neutral Sulfamethazine, which makes it a π-electron acceptor^27,28^. Raman spectra show that the degree of graphitization of modified activated carbon was higher where the graphitic structure of MAC can create additional π–π EDA interactions between SMX and MAC^29^.

In addition, the adsorption mechanism of Sulfamethazine on MAC is also affected by electrostatic interactions^30^ and coordination interactions^31^. The Sulfamethazine molecule can be hydrolyzed at different pH value and can assume several forms in an aqueous solution. At a pH < 5.0, however, the negative charge of the acidic oxygen functional groups demonstrates that there is a strong electrostatic interaction with the cationic center (–NH_3_^+^) of Sulfamethazine. The molecular structure of Sulfamethazine displays alkaline –NH_2_ and –OH groups that can coordinate with metal ions or generate hydrogen bonding. Many studies have proved coordination interactions between heavy metal ion and –NH_2_ and –OH^32,33^. Thus, it is extremely easy for Sulfamethazine to be adsorbed by solid particles and be removed from the aqueous solution. Generally, sulfonamides show good water solubility and exist mainly in the form of negative ions and neutral particles at neutral pH condition. Therefore, electrostatic interactions between Sulfamethazine and modified activated carbon are very weak. Therefore, the adsorption mechanism of Sulfamethazine on modified activated carbons is mainly micropore capture, hydrogen bonding interaction, and π-π EDA interaction. Electrostatic interaction and coordination interaction are also present under alkaline conditions.

## Conclusion

The surface area, total pore volume, micropore volume and mesopore volume of activated carbon were all increased to some extent after modification by Fe^3+^. The number of surface oxygenic functional groups of activated carbon was also increased, which can greatly improve the adsorption capacity. The Freundlich isotherm, Langmuir isotherm, and Temkin isotherm can provide satisfactory fits to the SMX adsorption data. The removal of SMX on MAC was significantly increased, and the maximum adsorption amount of Sulfamethazine on MAC was 17.2414 mg/g at 25 °C. However, the adsorption equilibrium time was almost invariant. The adsorption kinetics of Sulfamethazine on original and modified activated carbon are divided into fast and slow adsorption stages, and SMX adsorption was almost accomplished within 12 h rapidly. The pseudo-second-order kinetic model fits the SMX adsorption experimental data quite well. Intraparticle diffusion was not the only rate-controlling step of adsorption. The adsorption process was also affected by membrane diffusion and internal diffusion. The adsorption thermodynamic parameters showed that the adsorption process of the original and modified activated carbon was spontaneous and exothermic. A decrease in the temperature promoted the adsorption reaction. The pH value had a significant effect on the adsorption of SMX. The adsorption amounts of Sulfamethazine increased first and then decreased when pH was between 3 and 10. Micropore capture, electrostatic interaction, hydrogen bonding interaction, π-π EDA interaction and coordination interaction were the plausible mechanisms of adsorption.
